# Pitting Corrosion of Natural Aged Al–Mg–Si Extrusion Profile

**DOI:** 10.3390/ma12071081

**Published:** 2019-04-02

**Authors:** Quanmei Guan, Jing Sun, William Yi Wang, Junfeng Gao, Chengxiong Zou, Jun Wang, Bin Tang, Hongchao Kou, Haisheng Wang, Jianying Hou, Jun Gao, Jijun Ma, Jinshan Li

**Affiliations:** 1 CRRC Tangshan Co., LTD, Tangshan 063035, China; sjc-guanquanmei@tangche.com (Q.G.); sjc-sunjing@tangche.com (J.S.); wanghaisheng@tangche.com (H.W.); sjc-houjianying@tangche.com (J.H.); sjc-gaojun@tangche.com (J.G.); 2 State Key Laboratory of Solidification Processing, Northwestern Polytechnical University, Xi’an 710072, China; gaojunfeng25@foxmail.com (J.G.); zcx2016@mail.nwpu.edu.cn (C.Z.); nwpuwj@nwpu.edu.cn (J.W.); toby198489@163.com (B.T.); hchkou@nwpu.edu.cn (H.K.)

**Keywords:** pitting corrosion, natural aging, eddy current, 3D optical profilometry, high-throughput measurement

## Abstract

With the quick development of the high-speed railway and the service of the China Railway High-speed (CRH) series for almost a decade, one of the greatest challenges is the management/maintenance of these trains in environmental conditions. It is critical to estimate pitting damage initiation and accumulation and set up a corresponding database in order to support the foundations for interactive corrosion risk management. In this work, the pitting corrosion of a nature-aged commercial 6005A-T6 aluminum extrusion profile for 200 days was studied comprehensively. The heterogeneous microstructures were conventionally identified by the in situ eddy current, suggesting which investigated regions to fabricate samples for. After constant immersion for 240 h in 3.5 wt % NaCl, the shapes and depths of the pits were captured and measured by optical microscope (OM) and three-dimensional optical profilometry (OP), providing detailed quantification of uniform pitting corrosion. The typical features of the pits dominated by the distribution of precipitates include the peripheral dissolution of the Al matrix, channeling corrosion, intergranular attack, and large pits in the grains. Due to the high density of continuous anodic and cathodic particles constituted by alloying elements in coarse grains, the number of pits in the coarse grains was the highest while the number in the fine grains was the lowest, indicating that fine grains have the best corrosion resistance. The experimental dataset of the pit depth integrated with its corresponding microstructure would set the benchmark for further modeling of the pit depth and the remaining ductility, in order to manage the damage tolerance of the materials.

## 1. Introduction

Age hardenable Al-Mg-Si (6XXX) alloys are widely utilized in the aerospace, transport, automotive, and shipbuilding industries due to their high strength-to-weight ratio, recyclability, and resistance to corrosion [1,2,3]. They get further preference for industrial application than other series, such as Al-Cu-based (2XXX) and Al-Zn-based (7XXX) alloys, due to the lower amount of alloying element that they have and, thus, their lower cost [4]. Through well-known aging treatments, such as the T6 condition which is described by solid solution in the single phase region (520–540 °C) followed by quenching and artificially aged at a moderate temperature (150–200 °C), their mechanical properties can be modulated by the precipitation-hardening mechanism [4,5]. In general, the precipitation sequence of Al-Mg-Si alloys has been reported as [3,5,6,7,8]:

SSSS → solute clusters → GP Zones → β″→ β′

Here, SSSS represents the supersaturated solid solution. With the addition of Cu, the precipitation sequence changes to [7,9]:

SSSS → solute clusters → GP Zones → β″, L/S/C, QP, QC → β′, Q′→ Q

where L, C, S, and Q′ are metastable precipitates containing Cu. The L phase is believed to be a Q′ precursor, playing an important effect in strengthening the alloy [7].

Besides improving the mechanical properties of Al-Mg-Si alloys, the presence of Cu usually enhances precipitation-hardening kinetics, refines the microstructure, and reduces the negative effects of natural aging (NA) [2,7,10]. However, NA can significantly lessen the hardening kinetics and the maximum strength obtained in the subsequent artificial aging (AA) of the Al-Mg-Si alloy, which is the so-called negative effect of NA [5]. It has been reported that the negative NA effect is transient and reversed upon long storage times by the dominance of a process beneficial to precipitation in Al–Mg–Si alloys [3]. It is worth mentioning that several weeks or months are required to manufacture the final product from the raw Al alloys in transport and automobile industries [2,6]. Alloys are unavoidably stored for a period at room temperature, which undergo NA directly after quenching from solution heat treatment and result in two issues that need to be addressed, i.e., reduced formability and the negative NA effect [2,6]. For example, based on the investigations of an Al–Mg–Si alloy influenced by NA for 70 days and pre-treatment at 70 °C for 16 h prior to the AA at 175 °C, it was found that Mg–Si co-clusters formed during NA and small Guinier-Preston (GP) zones after pre-aging were present [8]. Si-rich clusters formed during NA can neither be dissolved nor grow during subsequent AA, which needs to be modified to enhance age-hardening behavior [6,11]. Similarly, AA7075 alloys naturally aged for 300 days were studied to reveal the effect of grain size on the pitting characteristics, showing that the frequency of the pitting current transients increased with the improved ratio of fine grains while the lifetime and the peak value of the current transient decreased [12].

The precipitates or second phases play an important role in the localized corrosion behavior of Al alloys, which is called pitting corrosion [13,14,15,16]. Pitting corrosion is one of the most destructive forms of corrosion that can lead to the catastrophic failure of structures/equipment, since corrosion damage is a frequent initiation site for cracks [17,18,19,20]. Pits can initiate at intermetallic particles resulting in microgalvanic interactions, so their size, quantity, location, continuity, and corrosion potential relative to that of the Al alloy matrix can influence pitting corrosion behavior [15,16,21]. Intermetallic compounds, including Al_2_CuMg, Al_3_Mg_2_, Mg_2_Si, and MgZn_2_, generally act as the anode and corrode preferentially with the surrounding Al matrix [16]. On the contrary, compounds such as Al_2_Cu, AlFeMnSi, AlCuFeMn, AlCuFeSi, and (Al, Cu)_x_(Fe, Mn)_y_Si are mostly cathode, resulting in the peripheral trenches of the Al matrix [16].

Since pitting corrosion is one of the most widespread and dangerous forms of localized corrosion in passive materials and is difficult to detect and predict [12], great efforts have been made to develop numerical models and reveal the fundamental mechanisms of pitting corrosion [18,19,20,22,23,24,25,26,27]. With the fast growth of the high-speed railway in China reaching to 3 × 10^4^ km in 2020 [28] and the service of the China Railway High-speed (CRH) series for almost a decade, one of the greatest challenges is the management and maintenance of these trains in environmental conditions. It is critical to estimate the initiation and propagation of pitting and set up a corresponding database in order to support the foundations for interactive corrosion risk management. Unfortunately, there is not much information/data showing the effect of NA on pitting corrosion and the mechanical properties of the Al–Mg–Si alloys utilized in high-speed rail. In this work, the pitting corrosion of a nature-aged commercial 6005A-T6 aluminum extrusion profile for 200 days was studied comprehensively via the top-down engineering approach [29]. The shapes and the depths of the pits after constant immersion for 10 days in 3.5 wt % NaCl were captured and measured by optical microscope (OM), three-dimensional optical profilometry (OP), and scanning electron microscope (SEM), providing detailed quantification of uniform pitting corrosion. Quantitative measurements of the depth of the pits are discussed in detail, providing a benchmark to develop numerical models and reveal the fundamental mechanisms of pitting corrosion.

## 2. Methodology

### 2.1. Materials and Sample Preparation

**Figure 1:**
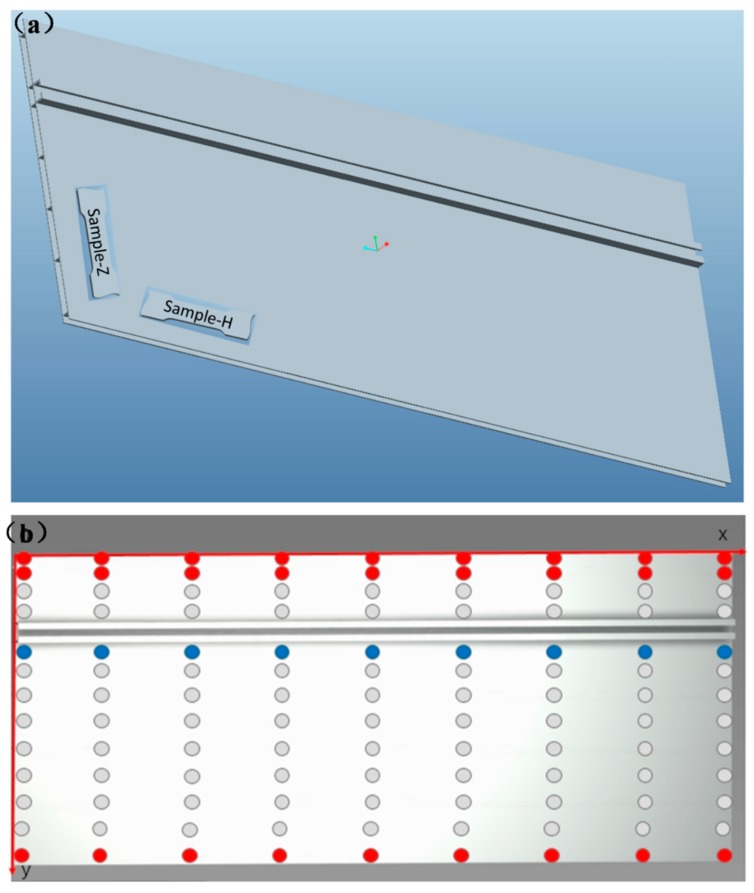
The schematic diagrams of the commercial 6005A-T6 extrusion profile for high-speed trains in different views: (**a**) 3D view and (**b**) top view. The fabricated tensile samples parallel and perpendicular to the extrusion direction are labeled by H and Z, respectively. The red, navy, and gray points highlight the selected region with the maximum, the normal, and the minimum value of the eddy current, respectively.

In this work, a commercial 6005-T6 extrusion profile for the frames of high-speed trains was selected as a case study, and its composition is listed in Table 1. It was nature-aged for 200 days at room temperature before completing the following measurements/investigations. The tensile specimens were fabricated based on the standard of TB/T 3260.1-2011 [30]; their schematic images are presented in Figure 1a. In line with standard of JB/T 7901-1999 [31], these tensile specimens were polished on SiC papers with mesh number of 240, 400, 800, and 1000 per square inch, followed by polishing with Buehler diamond paste (LIXIEYIQI, Zhejiang, China). Moreover, the pre-weighted tensile specimens endured a constant immersion, for 240 h, in 3.5 wt % NaCl aqueous solution at room temperature without adjusting the pH value, which has been a common approach for measuring pitting corrosion behavior [12,32] and meets the requirements of the recommended standard, JB/T7901-1999 [31]. The corrosion products were removed by rinsing in a solution consisting of 50 mL phosphoric acid, 20 g chromium trioxide, and 950 mL deionized water in an ultrasonic basin at a temperature range of 80–100 °C for 5–10 min. If the surface was not clear enough, specimens were cleaned in 7% HNO_3_ for about 10 s to remove any corrosion products [33,34].

Since the size of the extrusion profile utilized for a high-speed train is extremely large, the investigated regions were screened out through high-throughput eddy current measurements, as shown in Figure 1b. Here, the Sigma2008B eddy current conductivity meter (Ximen Tianyan Instrument Co., Ltd., Xiamen, China) was used, supporting the initial information of the structural heterogeneity. Subsequently, under the guidance of the recommended national standard (GB/T 3246.1-2000 [35]), the samples used for the microstructure characterizations were polished on SiC papers with mesh number of 80, 240, 400, 800, 1000, 1500, 2000, 2500, and 3000 per square inch, followed by polishing with Buehler diamond paste on a polishing cloth and subsequent electrochemical polishing. Finally, these specimens were rinsed with ethanol in an ultrasonic bath to remove any dust on the surface.

### 2.2. Surface Characterization and Pit Depth

Non-destructive OM measurements not only show the microstructures and morphologies of the surface but also provide a traditional approach for determining the pit depth after pitting corrosion. Here, the OLYMPUS GX51F optical microscope (Olympus Cop., Tokyo, Japan) and NPFLEX three-dimensional optical profilometry (Bruker NPFLEX, Tucson, AZ, USA) were utilized to present the pit depth, diameter, and volume for statistical analysis. In order to obtain precise data, the measurement of the pit depth must be carried out in triplicate (at least). Especially, when using the optical microscope to determine the pit depth under the recommended national standard, GB/T 18590-2001 [36], each pit is measured at four magnitudes of enlargement of ×100, ×200, ×500, and ×1000. Moreover, much more precise morphological parameters of a pit, including depth and volume, can be obtained by 3D optical profilometry with a depth resolution as high as 0.1 nm. The scanning electron microscopy (ZEISS GemniSEM500, Oberkochen, Germany) was performed using a beam energy of 15 kV to capture the secondary electron and backscattered electron images, thus revealing both the morphology and the compositions of the surface and precipitates/particles.

## 3. Results and Discussions

**Figure 2:**
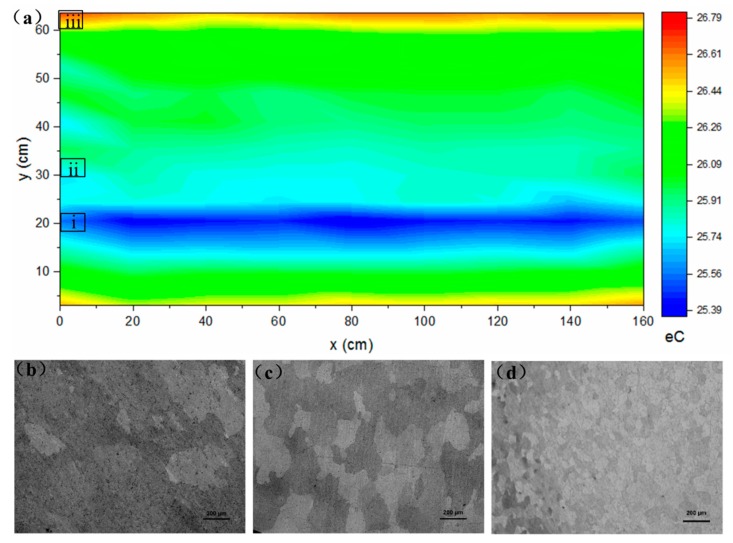
Eddy currents and classical optical microstructures of 6005A-T6 alloy. (**a**) The linear contour plot of the eddy current of the naturally aged 6005A-T6 alloy. (**b**–**d**) The screened-out microstructures with an eddy current of 25.58 MS/m, 26.24 MS/m, and 28.10 MS/m, which endured a constant immersion for 240 h in 3.5 wt % NaCl and are labeled as i–iii in (**a**), respectively. The maximum and minimum values of the eddy current are set to 26.79 MS/m and 25.39 MS/m in the red-green-blue (RGB) color scale in order to present the various zones clearly. The rectangles in (**a**) schematically highlight the positions of these three samples in (**b**–**d**), respectively.

To address the aforementioned challenge of screening out the investigated region in such a large extrusion profile shown in Figure 1, high-throughput eddy current measurements were performed to identify the structural heterogeneity and, thus, to fabricate the specimens. Figure 2a shows the linear contour plot of the eddy current of the naturally aged 6005A-T6 alloy. Based on the initial rough measurements, it was found that the microstructures along the x-direction were homogeneous. On the contrary, the microstructures along the y-direction were heterogeneous, caused by the difference in the shapes and size of the extrusion profile shown in Figure 1a. This is the reason why the high-throughput measurements, with a step of 20 cm along the x-direction and ~5 cm along the y-direction, were performed. It is also suggested that different microstructures could be conventionally captured if the specimens were manufactured along the y-direction, which is perpendicular to the extrusion direction. Accordingly, the screened-out microstructures with an eddy current of 25.58 MS/m, 26.24 MS/m, and 28.10 MS/m, are presented in Figure 2b–d. The groove position had the largest grain size while the edge part of the profile had the smallest grain size. Moreover, with a reduction in grain size, the eddy current enhanced. It is worth mentioning that these microstructures all endured a constant immersion for 240 h in 3.5 wt % NaCl aqueous solution. The number of pits in the large grains was the highest while the number in the fine grains was the lowest, indicating that fine grains have the best corrosion resistance in the Al matrix. This is because there are more intermetallic particles in coarse grains, which are also bigger and have more continuous anodic and cathodic particles constituted by alloying elements [12].

**Figure 3:**
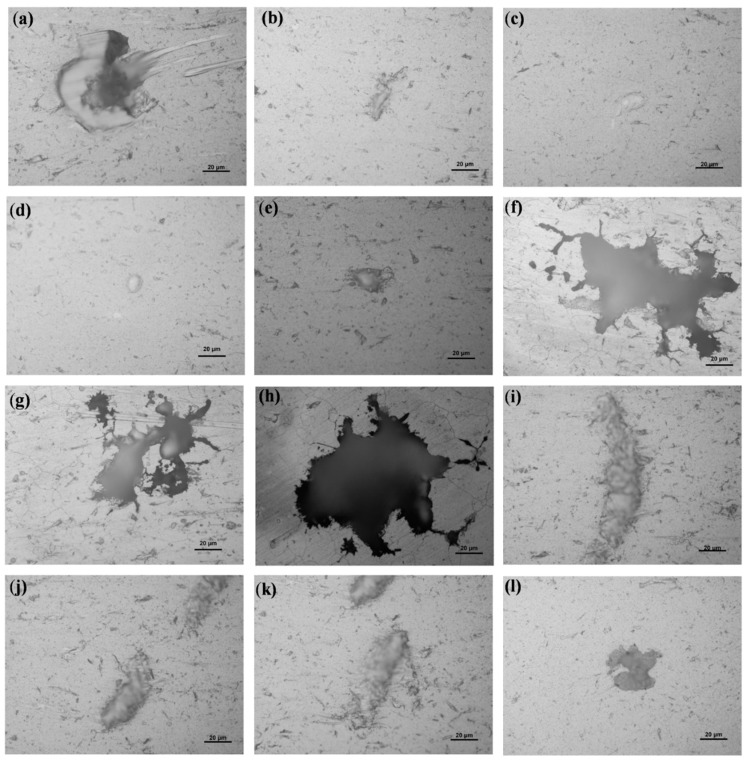
Optical morphologies of pits observed on Sample-H after constant immersion. (**a**–**l**) are the selected pitting points from A to K listed in Table 2, respectively.

**Figure 4:**
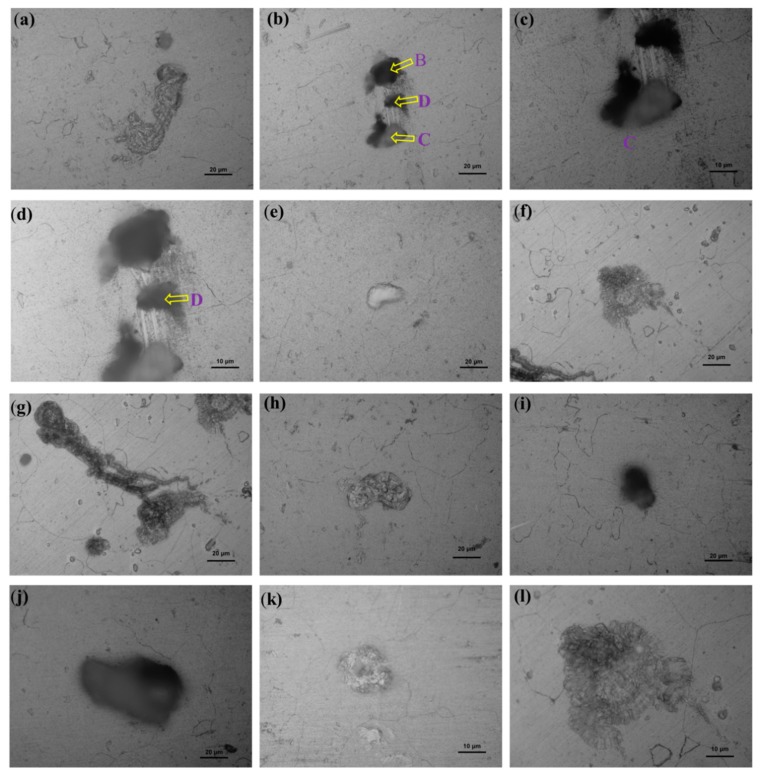
Optical morphologies of pits observed on Sample-Z after constant immersion: (**a**–**k**) are the selected pitting points from A to K listed in Table 2, and (**l**) is the amplified region of (**f**), highlighting the caved grains along the grain boundaries.

**Figure 5:**
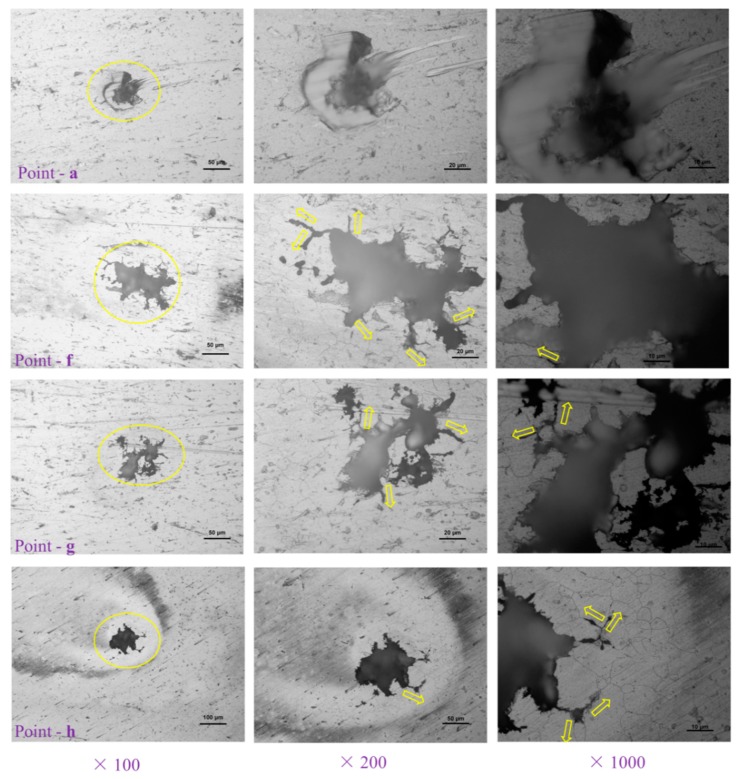
Morphologies of classical pits at various magnitudes of enlargement during measuring of their depths, the data of which are listed in Table 2. The yellow arrows highlight the initiation of cracks within the pit-to-crack transformation scheme.

Figure 3 and Figure 4 display the classical morphologies of the pits of Sample-H and Sample-Z after constant immersion, respectively. It is well known that the classical morphologies of precipitates of 6XXX alloys are the plate-like β-Al_5_FeSi and the rounded α-Al_12_(Fe, Mn)_3_Si particles ranging between 1 and 10 um [37]. The needle-like β″ phase, which is normally identified as Mg_5_Si_6_, has been considered to be the most effective strengthening precipitate among all kinds of precipitates in 6XXX alloys [5]. The evolution of the intermetallic structures of β-Al_5_FeSi and α-Al_12_(Fe, Mn)_3_Si have been captured in 3D, revealing qualitative and quantitative analysis of the reconstructed morphology of the metallic microstructures of 6005A alloy, especially the connectivity/distribution of those intermetallics [38]. Based on the morphologies of those pits, it is revealing that the precipitates play an extremely important role in pitting corrosion by acting either as a cathode or anode resulting in the dissolution of themselves or the Al matrix, shown in Figure 3 and Figure 4. The typical features of pitting corrosion include: (i) peripheral dissolution/trenching of the Al matrix around the small size cathodic precipitates, (ii) channeling corrosion, (iii) intergranular attack initiating the pitting, and (iv) large pits in the grains. As shown in Figure 5, several large corrosion pits in Figure 3 were chosen and enlarged at different magnitudes, revealing that the front of the corrosion pathways were dominated by grain boundaries due to intergranular corrosion and indicating the initiation of cracks within the pit-to-crack transformation scheme.

Moreover, it is worth mentioning that the polishing pit should be avoided in the measurement of pit depth, because its morphology is different to the etching one in size and depth. In particular, the depth of the etching pit is measured at three magnitudes of enlargement under the guidance of the recommended national standard GB/T 18590-2001 [36]. The strategy for selecting these magnitudes of enlargement is dominated by the volume and depth of the pits. In general, the larger the pit, the smaller the magnitude of enlargement (×100). As listed in Table 2, the depths of each point shown in Figure 3 and Figure 4 are summarized. For large pits, it is not necessary to measure the pit depth via the highest magnitude of enlargement (×1000). For a small pit, the lowest magnitude of enlargement (×100) should be avoided. After measuring each pit depth three times, the average value can be estimated for each pit. The corrosion pits with a depth about 10 μm have the most uniform morphology for those selected regions together with the deepest ones of 79.7 μm and 30.3 μm on the sample fabricated parallel and perpendicular to the extrusion’s direction, respectively. Similarly, the unique depth of the corrosion pit of 7075-T6 alloy was 94 μm after a constant immersion in NaCl for 30 days at pH 5 and 20 °C [32]. Since the pit depth will increase with immersion time [34], it is critical to set up a corresponding database for the further modeling of pit depth and the accuracy of the predicted results, such as the evolution of pit depth, which is critically important to the damage tolerance of the materials.

**Figure 6:**
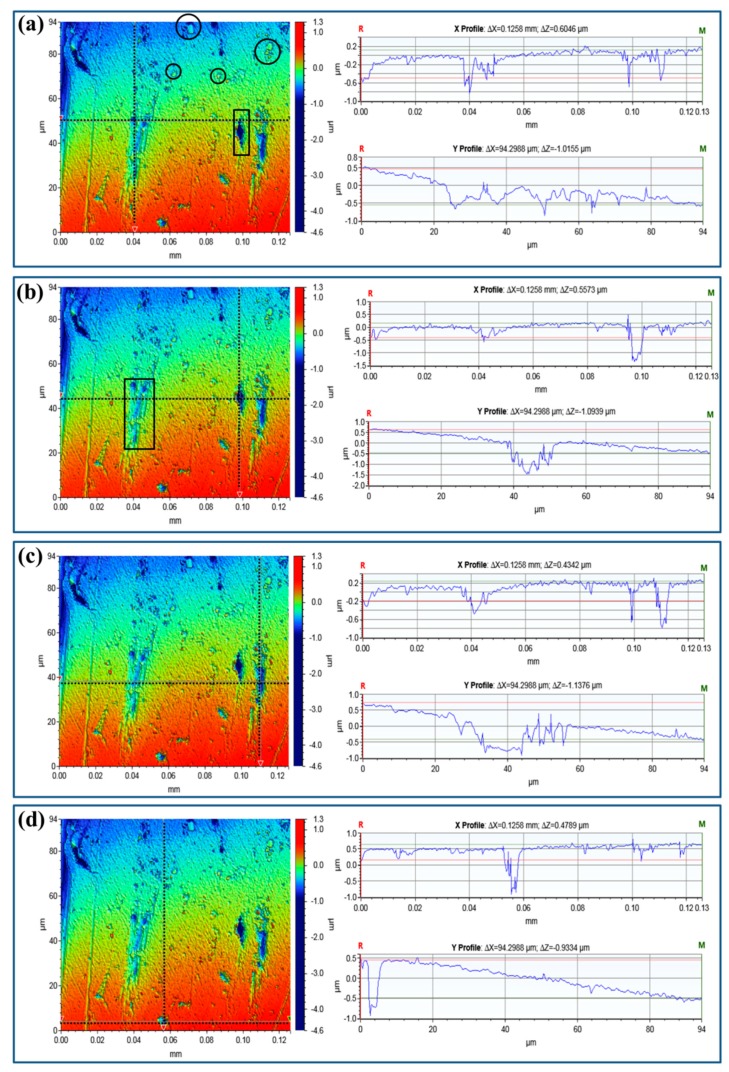
Three-dimensional optical profilometry images of Sample-H after constant immersion for 240 h in 3.5 wt % NaCl together with the line profiles of the pits. The black circles highlight the peripheral dissolution/trenching of the Al matrix around the cathodic precipitates, while rectangles show the classical channeling corrosion merging pits. (**a**–**d**) highlight the line profiles crossing the selected pit, which are identified these black dot lines.

**Figure 7:**
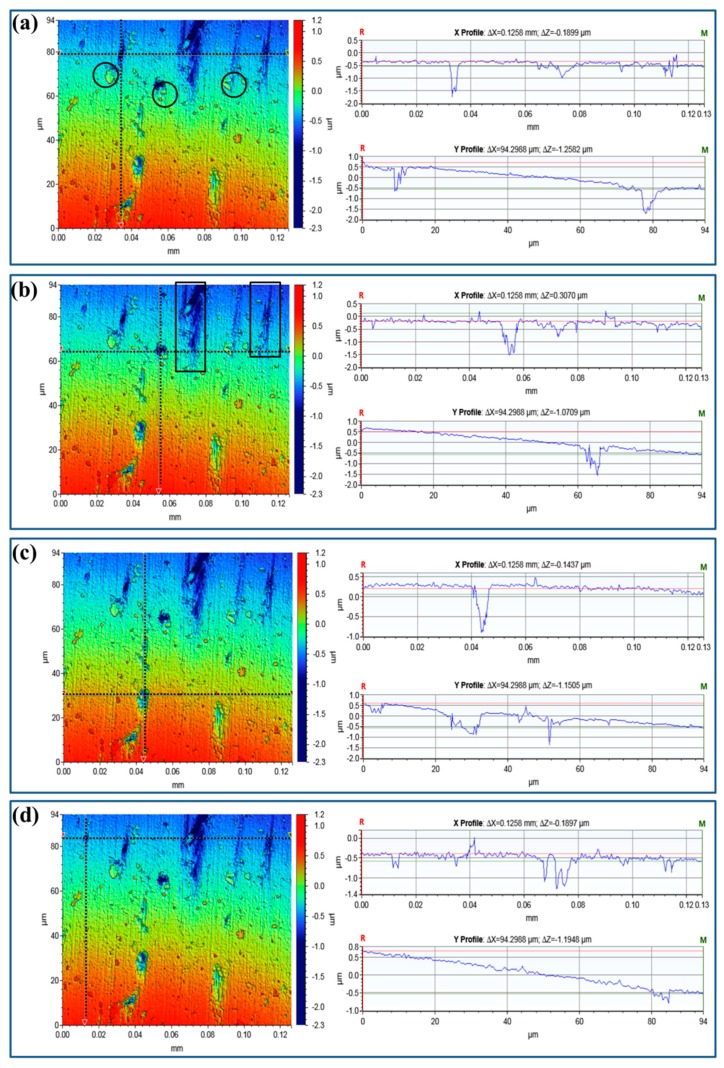
Three-dimensional optical profilometry images of Sample-Z after constant immersion for 240 h in 3.5 wt % NaCl together with the line profiles of the measured pits. (**a**–**d**) highlight the line profiles crossing the selected pit, which are identified these black dot lines.

Besides the surface morphology, three-dimensional optical profilometry is usually used to analyze the number, size, and depth of pits after a constant immersion test [32,34]. Figure 6 and Figure 7 present the qualitative and quantitative descriptions of the pits of Sample-H and Sample-Z, respectively. Due to the different heights among the pits, precipitates, and the matrix, the samples are characterized clearly by the gradient colors scaled depths. In particular, the black circles in Figure 6a, Figure 7a highlight the aforementioned peripheral dissolution/trenching of the Al matrix around the small cathodic precipitates. These narrow blue stripes, highlighted by rectangles, show classical channeling corrosion, which also merges some of the pits. In those selected regions, the depths of the coarse pits can be measured accurately, shown as linear profiles in Figure 6 and Figure 7.

**Figure 8:**
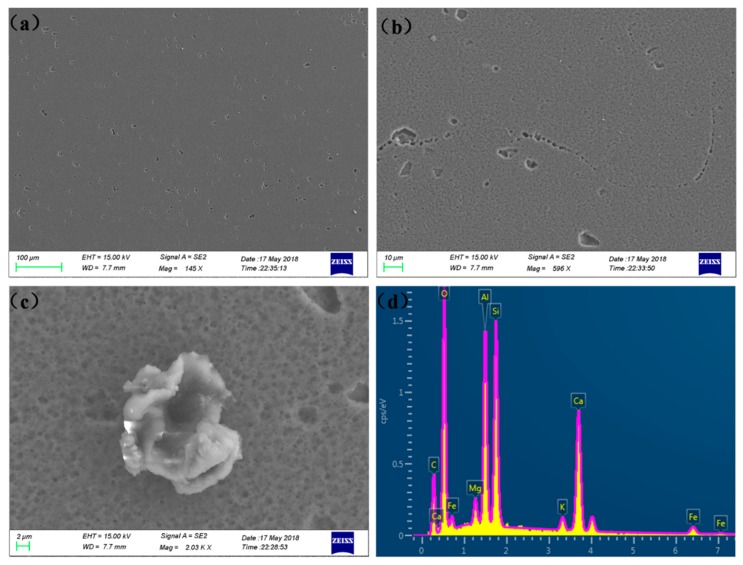
SEM images of the surface of 6005A-T6 alloy after constant immersion for 240 h in 3.5 wt % NaCl: (**a**) the morphology of the pits, (**b**) the etched grain boundaries enriched with pits, and (**c**,**d**) the morphology of the bright particle together with its EDS analysis.

Figure 8 presents the SEM images of the surface of the 6005A-T6 alloy after constant immersion for 240 h in 3.5 wt % NaCl. Three typical structures, including the morphology of the pits, the etched grain boundaries enriched with pits, and the corrosion product, are displayed in Figure 8a–c, respectively. The morphologies of the pits are typically dominated by the distribution of precipitates, such as the highlighted zones in Figure 6a, Figure 7a. Based on the Energy Dispersive Spectrometer (EDS) analysis of the composition of the particle shown in Figure 8c,d, it can be seen that the corrosion product is enriched with Al, Si, O, and Fe. It is worth mentioning that solute clustering and precipitates during natural aging play key roles not only in solid-solution strengthening but also in pitting corrosion.

On one hand, the developed heterogeneous structures constructed by a series of hardening precipitates and solute clusters result in precipitation strengthening and solid-solution strengthening mechanisms [1,33,39]. For example, the purpose of the T6 treatment (or peak aging) of the 6005A-T6 alloy is to obtain a high number density of precipitates, giving maximum strength, and it is commonly utilized in the processing of high-strength Al alloys [1]. Moreover, the heterogeneous solute distribution has a strong effect on the dislocation motion (i.e., pinning effect), thus, strengthening the materials.

On the other hand, heterogeneous structures constituted of a series of hardening precipitates and solute clusters will initiate localized corrosion at the secondary phases or their surrounding Al matrix, which will form pits and begin the degradation of the mechanical properties of the alloy. In general, solute clustering during natural aging is mainly dominated by frozen-in excess vacancies, which could be suppressed by a short-term interruption of the quenching process [40]. Precipitates usually have a composition different from that of the matrix, yielding a local chemistry change [1]. The corresponding electrochemical properties, including pitting corrosion and stress corrosion cracking, are detrimentally affected by precipitate-free zones [1]. Typically, iron-rich particles have higher cathodic activities than pure Al, meaning that pitting initiations prevail at cathodic (Cu- or Fe-rich) particles in chloride containing neutral aerated solution [12,41]. Oxygen reduction is the dominant reaction and is conventionally carried out on these precipitate particles [12,41,42]. This is the reason why our captured corrosion product is enriched in Al, Si, O, and Fe, as presented in Figure 8d. Moreover, the morphology of the Al(Fe, Cr)Si particles of 6005A alloy could be optimized from granular to rod by increasing the aging temperature or aging time, which could also be segregated at the grain boundaries [43]. Therefore, through combining the information from Figure 8d, it is understood that the arrows in Figure 5 indicate that the segregation of Fe and Si and the precipitation of Al(Fe, Cr)Si particles contribute to the aforementioned intergranular attack, initiating the pitting.

Motivated by the management/maintenance of the China Railway High-speed series in environmental conditions, it is expected that more efforts will be performed to estimate pitting damage initiation and propagation, to set up a corresponding database, and to develop advanced processing strategies, which will support the foundations for interactive corrosion risk management. The measured pit depth would not only help to understand the effective thickness/cross-section area of the specimens but also to create models that predict the remaining stress and to manage the damage tolerance of the materials. For example, it has been proposed that the typical stress reduction for the pre-corroded tensile specimens at different immersion times can be expressed as Δσ = σ_0_ – σ_j_ [44]. The ratio of remaining stress (σ_j_) to benchmark stress (σ_0_) is assumed to be equivalent to the ratio of remaining undamaged cross-section area (A_j_) to original section area (A_0_) [44,45]. Moreover, based on the well-known negative natural aging (NA) effect, it has been found that hardness recovery in the nugget zone and the thermo-mechanically affected zone of 6005A-T6 alloy have been observed during post-weld NA, which attributes to the formation of clusters at an early stage and GP-zone precipitation after 4 weeks of NA [46]. Large and deep pits are replaced with small and shallow ones after equal-channel angular pressing in 6061 alloy, which leads to a substantial increase in the dislocation density, re-distribution of precipitates, crystallite refinement, alteration of the surface area of cathodic sites, and increase in the volume fraction of grain boundaries that affects corrosion resistance [47]. In order to support the foundations for the interactive corrosion risk management of equipment/infrastructure, such as the aforementioned trains in environmental conditions, more efforts are required to estimate pitting damage initiation and accumulation, set up the corresponding database, and develop advanced processing strategies.

## 4. Conclusions

In this work, the pitting corrosion of a nature-aged commercial 6005A-T6 aluminum extrusion profile for 200 days was studied comprehensively. To address the challenge of screening out the investigated region in such a large extrusion profile, high-throughput eddy current measurements were performed to identify the aforementioned structural heterogeneity, in order to fabricate the specimens. After constant immersion for 240 h in 3.5 wt % NaCl, it was found that the traditional features of pitting corrosion include: (i) peripheral dissolution/trenching of the Al matrix around the small size cathodic precipitates, (ii) channeling corrosion, (iii) intergranular attack initiating the pitting, and (iv) large pits in the grains. Due to the high density of continuous anodic and cathodic particles constituted by alloying elements in coarse grains, the number of pits in the coarse grains was the highest while the number in the fine grains was the lowest, indicating that fine grains have the best corrosion resistance. The experimental dataset of pit depth integrated with the corresponding microstructures would set the benchmarks for further modeling of pit depth and the accuracy of the predicted results, such as the evolution of the pit depth and the remaining ductility, in order to manage the damage tolerance of the materials.

## Figures and Tables

**Table 1:** Chemical composition of the commercial 6005A-T6 alloy at manufacture (wt %).

Elements	Si	Fe	Cu	Mn	Mg	Cr	Zn	Ti	Al
**Content**	0.5–0.9	≤0.35	≤0.30	≤0.30	0.40–0.7	≤0.30	≤0.20	≤0.10	Bal.

**Table 2:** Depth of pits measured by optical microscope.

Sample	Pit No.	The Depth of Pit at Magnitudes of Enlargement				Averaged Depth (μm)
		×1000	×500	×200	×100	
Sample-H	A		18	20	17	18.3
	B	6	5	5		5.3
	C	3	3	5		3.7
	D	4	4	4		4.0
	E	17	12	12		13.7
	F		69	72	70	70.3
	G		39	41	40	40.0
	H		79	80	80	79.7
	I	9	11	10		10.0
	J	9	8	9		8.7
	K	10	8	12		10.0
	L	12	11	12		11.7
	M	6	6	6		6.0
Sample-Z	A	4	5	5		4.7
	B	8	9	9		8.7
	C	13	10	12		11.7
	D	9	8	7		8.0
	E	6	6	6		6.0
	F	4	4	5		4.3
	G		4	4	4	4.0
	H		31	30	30	30.3
	I		12	13	12	12.3
	J	12	11	11		11.3
	K	2	2	2		2.0
	L	6	5	7		6.0
